# Sense of Unity and Self-Reported Health Among 15-year-Olds: Findings From the Swedish 2017/18 Health Behavior in School-Aged Children Study

**DOI:** 10.3389/ijph.2021.621964

**Published:** 2021-04-20

**Authors:** Joakim Wahlström, Bitte Modin, Johan Svensson, Petra Löfstedt, Sara Brolin Låftman

**Affiliations:** ^1^ Department of Public Health Sciences, Centre for Health Equity Studies (CHESS), Stockholm University, Stockholm, Sweden; ^2^ Department of Public Health Sciences, Centre for Social Research on Alcohol and Drugs (SoRAD), Stockholm University, Stockholm, Sweden; ^3^ Department of Public Health and Community Medicine, Sahlgrenska Academy, University of Gothenburg, Gothenburg, Sweden

**Keywords:** sense of unity, self-reported health, health complaints, self-rated health, adolescents

## Abstract

**Objectives:** Sense of unity refers to the positive feeling of being part of a larger social structure. This study aimed to investigate to what extent adolescents report sense of unity and if this differs across groups, and to assess the associations between sense of unity and self-reported health while taking into account sociodemographic characteristics and tangible social relationships.

**Methods:** Data were obtained from the 2017/18 Swedish Health Behavior in School-aged Children study, using information collected among 15-year-old students (*n* = 1,392). Linear and binary logistic regression analyses were performed.

**Results:** The participants reported overall high levels of sense of unity. Sense of unity did not differ by gender, but adolescents without an immigrant background and those with higher family affluence reported higher levels. Sense of unity was inversely associated with psychological complaints, somatic complaints, and less than good self-rated health, even when adjusting for sociodemographic characteristics and family, classmate, and teacher relationships.

**Conclusion:** This study suggests that sense of unity may be an important social determinant for adolescent health. More research is needed on the origins and implications of sense of unity.

## Introduction

Adolescence has been recognized as a period that provides “a critical opportunity for prevention and intervention to support young people’s healthy growth and development, promote future health and wellbeing in adulthood, and, as such, underpin the health of the next generation” [1]. Although the adolescent years are generally characterized by few physical health problems, self-reported psychological and somatic health complaints are common [2], and a non-negligible minority of adolescents rate their general health as less than good [3–6]. Such health problems reflect adolescents’ lacking opportunities to achieve their full current health potential [6], but are also associated with increased risks of adverse health in adulthood [7–9]. Accordingly, identifying the social determinants of adolescents’ self-reported health is a relevant task. As highlighted by Viner et al. [10], social factors that affect adolescents’ health exist at personal, family, community, and national levels.

At the personal level, belongingness has been identified as a basic human need and thus constitutes an important factor for health and wellbeing [11]. Sense of belonging refers to an individual’s feelings of being valued and needed and the perception of fitting into the environment [12]. General sense of belonging has been studied in relation to mental health outcomes among adults, and to a limited extent also among adolescents, with results showing that general sense of belonging predicted adolescents’ depressive symptoms [13, 14]. On the whole, however, research into young people’s feelings of belongingness to society at large and the links with health is scarce. To gain further knowledge about this topic, we will make use of the newly formulated concept of sense of unity, which was recently developed by scholars in the Health Behavior in School-aged Children (HBSC) International Research Network, and its complementary scale aiming to capture sense of unity among adolescents [15].

### Sense of Unity

Sense of unity refers to the positive feeling of being part of a larger social structure. A high sense of unity, indicated by strong feelings of interdependence, belonging, trust, and selflessness in relation to groups and communities in a general sense, is believed to provide adolescents with a sense of common good as well as to promote positive development and foster mental health [15].

The fundamental human need to belong is expressed through the intrinsic motivation of forming and maintaining of interpersonal bonds [11]. While social bonds are mostly conceptualized as resources that are external to the individual, such as social networks and social support [16], sense of unity instead refers to personal assets in terms of the individual’s feelings of belongingness to society. These feelings are however likely to largely be influenced by his or her experiences of social relationships. Accordingly, strong and supportive interpersonal bonds in specific contexts can be assumed to promote an individual’s sense of unity.

Besides sense of belonging, sense of unity also has explicit similarities with two other concepts that stem from different fields of health-related research, namely sense of community and connectedness [15]. Sense of community was described in Sarason’s seminal work as the perception of similarity to, and interdependence with, others, wanting to maintain interdependence through reciprocity and the feeling of being part of a larger stable structure [17]. McMillan and Chavis [18] further defined sense of community as consisting of four parts; membership, influence, integration and fulfilment of needs, and shared emotional connection. Together, these parts bring together a feeling of belonging to a group where members matter to each other and that individuals’ needs will be met through acts from the group. Importantly, sense of community is concerned with a specific community, which may be based on locality or on special interests (e.g., professional, spiritual, etc.) [18]. Sense of community has been found to be a predictor of social well-being among adolescents [19, 20]. Connectedness is not as clearly defined and has been conceptualized in a multitude of ways in prior research, e.g. the quality of a relationship, the degree of liking an environment or relationship, and the possession of feelings or attitude states [21]. Specifically, relationships in different domains, e.g. family, peers, and school, have been in focus. Despite the variation in the definitions and operationalizations of connectedness, studies have consistently shown clear links between connectedness and health outcomes, among adolescents, with connectedness thought of as being a protective factor for ill health [22–24]. Although sense of unity has similarities with both sense of community and connectedness, there are also differences between these concepts. While sense of community refers to feelings of being part of a specific community, and connectedness often refers to the properties of tangible social relationships, sense of unity represents an individual’s feelings of belonging to society in a wider, more general sense. Yet, as mentioned above, and as indicated by previous research on the associations between social support in specific domains and school sense of community [25], it seems plausible that positive social relationships, e.g., in the family and at school, will promote an individual’s sense of unity. Other potential determinants of sense of unity include sociodemographic characteristics such as socioeconomic position and migrant background, both of which have been found to be linked to sense of community and connectedness [23, 26–28].

### The Current Study

Drawing on a sample of Swedish 15-year-olds, this study examines the associations that sense of unity shares with psychological and somatic complaints and self-rated health.

Prior research has shown that girls are more likely to report adverse health than boys [29]. There is also evidence of an inverse association between family socioeconomic position and adolescent health [29, 30], although this may vary by, e.g., the measure of socioeconomic position as well as across health outcomes [31, 32]. With regards to the relationship between migration background and adolescent health, findings are mixed [29, 33, 34]. Furthermore, students’ assessments of their relationships with family, classmates, and teachers have been shown to be linked with adolescent health in prior studies [35, 36], and are assumed to be associated with sense of unity as well.

An individual’s sense of unity, health, and perceived social support are likely to be mutually reinforcing, meaning that any associations between these variables probably are bi-directional. To disentangle the direction of any causal relationship is beyond the scope of the current study. However, guided by the conceptual similarities of sense of unity with sense of community and connectedness and previous empirical findings regarding the connections between these concepts and adolescent health, in the regression analyses we treat sense of unity as the independent variable, psychological and somatic complaints and self-rated health as the dependent variables, and social relationships as confounding variables.

The aims of this study were, firstly, to investigate the extent to which adolescents report sense of unity; secondly, to examine differences in sense of unity by sociodemographic background characteristics and by family, classmate, and teacher relationships; and thirdly, to assess the associations between sense of unity and psychological complaints, somatic complaints, and self-rated health while also adjusting for sociodemographic background characteristics and family, classmate, and teacher relationships.

## Methods

### Data Material

The data were obtained from the Swedish Health Behavior in School-aged Children (HBSC) 2017/18 survey, including students in grades 5, 7, and 9 (ages 11, 13, and 15). Statistics Sweden performed the sampling and the data collection. A two-step cluster sampling design was used for each grade. First, a random, nationally representative sample of schools was drawn, and thereafter, one class in each school that had agreed to participate was randomly selected. Of the 450 schools that were included in the sampling frame, 213 agreed to participate, i.e., 47% [37]. In total, 4,294 students participated, corresponding to 89% of the students in the schools that had agreed to take part [38]. Since the measure of sense of unity was only included in the questionnaires for the oldest students (15-year-olds), the current study covers this age group (*n* = 1,661; response rate 87%). After exclusion of cases due to internal non-response (*n* = 269), the study sample consisted of 1,392 students distributed across 78 classes (84% of the total sample). Further information about the international HBSC study is provided elsewhere [1, 2].

### Measures

#### Dependent Variables

Psychological complaints were constructed from four items: “In the last 6 months: how often have you had the following….?”: “Feeling low”; “Irritability or bad temper”; “Feeling nervous”; “Difficulties in getting to sleep.” Response alternatives were: 1) “About every day,” 2) “More than once a week,” 3) “About every week,” 4) “About every month,” and 5) “Rarely or never.” Internal consistency was good (Cronbach’s α = 0.70). Based on these four items, an index ranging between 4–20 was created, with higher values indicating higher levels of psychological complaints [39].

Somatic complaints were constructed from four items: “In the last 6 months: how often have you had the following….?”: “Headache”; “Stomachache”; “Backache”; “Feeling dizzy.” The response alternatives were the same as for psychological complaints. Internal consistency was acceptable (Cronbach’s α = 0.60). An index ranging between 4–20 was created, with higher values indicating higher levels of somatic complaints [39].

Self-rated health was captured by the question: “Would you say your health is … … ?.” Response alternatives were 1) “Excellent,” 2) “Good,” 3) “Fair,” and 4) “Poor.” Those who answered “fair” or “poor” were categorized as having less than good self-rated health. This categorization is commonly used in studies of self-rated health among both adults [40] and adolescents [4, 5].

#### Independent Variable

Sense of unity was constructed from eight items: “I feel … ”: “a strong sense of togetherness”; “responsibility for others”; “that it is good to be part of a community”; “that I contribute without expecting anything in return”; “that I mean a lot to others”; “that others mean a lot to me”; “that I trust most people”; “that we rely upon each other.” The response alternatives were 1) “Not at all true for me,” 2) “Not very true for me,” 3) “Neither true nor untrue for me,” 4) “Not at all true for me,” and 5) “Really true for me.” The scores were summed to an index ranging from 8 to 40, with higher values indicating higher sense of unity. For individuals included in the study sample with missing values on at most two items (*n* = 106), the missing values were replaced with the individual mean for the remaining items. Internal consistency was high (Cronbach’s α = 0.87, boys: 0.90; girls: 0.84). Respondents with at least one missing value on these questions were more likely to rate their relationships with teachers as relatively poor compared to respondents without missing values, otherwise no statistically significant difference was detected between the two groups (data not presented). Due to the skewed distribution, with a majority of respondents reporting a high sense of unity, the index was divided into three categories of about equal size indicating high (scores 35–40), medium (scores 31–34), and low (scores 8–30) sense of unity. More information about the instrument is found elsewhere [15].

#### Control Variables

Gender included two categories: boys and girls.

Immigrant background distinguished between participants born in Sweden to (at least one) parent(s) born in Sweden, and those born abroad or born in Sweden with two parents born abroad.

Socioeconomic position was based on the *Family Affluence Scale* (FAS), which reflects household expenditure and consumption. FAS consists of six items: number of household computers; number of household cars; having an own bedroom; number of holidays abroad last year; if the household owns a dishwasher; number of household bathrooms [41]. The scores on the six items were summed up to an index with the possible range 0–13. The index was divided into three categories of about equal size indicating (relatively) high (scores 11–13), intermediate (scores 9–10), and low (scores 2–8) family affluence.

Family relationships were constructed from four questions: “My family really tries to help me,” “I get the emotional help and support I need from my family,” “I can talk about my problems with my family,” and “My family is willing to help me make decisions.” Response options ranged from 1 (“Very strongly disagree”) to 7 (“Very strongly agree”). Internal consistency was high (Cronbach’s α = 0.90). The scores were summed to an index ranging from 4 to 28 and subsequently divided into three categories of about equal size indicating (relatively) strong (score 28), intermediate (scores 24–27), and poor (scores 4–23) family relationships.

Classmate relationships were measured from three questions: “The students in my class (es) enjoy being together,” “Most of the students in my class (es) are kind and helpful,” and “Other students accept me as I am.” Response options ranged from 1 (“Strongly agree”) to 5 (“Strongly disagree”). Internal consistency was high (Cronbach’s α = 0.82). The scores were summed to an index ranging from 3 to 15 and subsequently divided into three categories of about equal size indicating (relatively) strong (scores 13–15), intermediate (scores 11–12), and poor (scores 3–10) classmate relationships.

Teacher relationships were constructed from three questions: “I feel that my teachers accept me as I am,” “I feel that my teachers care about me as a person,” and “I feel a lot of trust in my teachers.” Response options and the index range were the same as for classmate relationships. Internal consistency was high (Cronbach’s α = 0.87). The scores were summed to an index ranging from 3 to 15 and subsequently divided into three categories of about equal size indicating (relatively) strong (scores 14–15), intermediate (scores 11–13), and poor (scores 3–10) teacher relationships.

For individuals included in the study sample with missing answers on, at most, one item per relationship measurement (*n* = 4–21), the missing values were replaced with the individual mean for the remaining items.

### Statistical Methods

Differences in the proportion of respondents who reported high, medium, and low sense of unity by gender, immigrant background, socioeconomic position, and family, classmate, and teacher relationships were examined by means of chi2-tests. To assess the associations between sense of unity and psychological and somatic complaints, linear (OLS) regression analysis was used, whereas binary logistic regression was used in the analyses of self-rated health. Since the students were clustered in school classes, robust standard errors were estimated with Stata’s “cluster” command. First, a series of crude analyses was performed, testing one independent variable at a time. In Model(s) 1, we adjusted for gender, immigrant background, and socioeconomic position. In Model(s) 2, family, classmate, and teacher relationships were added as control variables. Interaction terms between the trichotomized sense of unity measure and each of the control variables, were then added to Model(s) 2 to examine if any interaction effects could be detected. Finally, a series of sensitivity analyses was performed to check the robustness of the results.

## Results

Descriptive statistics of the study variables are presented in Table 1. The mean values for psychological and somatic complaints were 11.3 and 8.8 respectively, while 8.6% reported less than good self-rated health. As described above, the sample was divided into three groups, capturing students whose sense of unity was, relatively, high (36.7%), medium (30.7%), and low (32.6%). The study sample consisted of a somewhat larger proportion of girls (53.6%) and a majority did not have an immigrant background (78.7%). The distributions of our trichotomized measures of socioeconomic position as well as of family, classmate, and teacher relationships are presented at the bottom of Table 1.

**TABLE 1:** Descriptives. *n* = 1,392. Health Behavior in School-aged Children study, Sweden, 2017/18.

	Mean	s.d
Psychological complaints	11.3	0.11
Somatic complaints	8.8	0.10
	n	%
Self-rated health		
Very good or good	1,272	91.4
Less than good	120	8.6
Sense of unity		
High	511	36.7
Medium	427	30.7
Low	454	32.6
Gender		
Boys	646	46.4
Girls	746	53.6
Immigrant background		
No	1,096	78.7
Yes	296	21.3
Socioeconomic position (FAS)		
High	426	30.6
Medium	579	41.6
Low	387	27.8
Family relationships		
Strong	423	30.4
Intermediate	505	36.3
Poor	464	33.3
Classmate relationships		
Strong	495	35.6
Intermediate	532	38.2
Poor	365	26.2
Teacher relationships		
Strong	418	30.0
Intermediate	565	40.6
Poor	409	29.4

To examine the associations between sense of unity and the sociodemographic characteristics and family, classmate, and teacher relationships, cross tabulations with chi2-tests were performed, with results presented in Table 2. There was no statistically significant gender difference in sense of unity. However, adolescents with an immigrant background reported lower sense of unity compared with those without such a background, and adolescents with a lower socioeconomic position reported lower sense of unity compared with those with a higher socioeconomic position. Higher assessments of family, classmate, and teacher relationships were associated with a higher sense of unity.

**TABLE 2:** Sense of unity by sociodemographic characteristics and different types of social relationships. Per cent. *n* = 1,392. Health Behavior in School-aged Children study, Sweden, 2017/18.

	Sense of unity			χ^2^
	Low	Medium	High	
Gender				
Boys	34.5	28.3	37.2	
Girls	31.0	32.7	36.3	3.57
Immigrant background				
No	30.7	31.6	37.8	
Yes	39.9	27.4	32.8	8.99*
Socioeconomic position (FAS)				
High	23.7	27.7	48.6	
Medium	31.4	33.2	35.4	
Low	44.2	30.2	25.6	58.62**
Family relationships				
Strong	19.2	22.0	58.9	
Intermediate	28.1	36.6	35.3	
Poor	49.8	32.1	18.1	185.77***
Classmate relationships				
Strong	18.8	26.5	54.8	
Intermediate	32.0	35.9	32.1	
Poor	52.3	28.8	18.9	159.03***
Teacher relationships				
Strong	21.3	23.0	55.7	
Intermediate	28.0	36.1	35.9	
Poor	50.6	31.1	18.3	153.29***

***p < 0.001 **p < 0.01 *p < 0.05.

The analyses of sense of unity and psychological complaints are presented in Table 3. The crude estimates revealed a gradient pattern: compared with adolescents who reported a high sense of unity, higher levels of psychological complaints were shown for those with a medium sense of unity (*b* = 1.68, *p* < 0.001) and for those with a low sense of unity (*b* = 2.90, *p* < 0.001). These estimates remained strong and statistically significant when adjusting for sociodemographic characteristics in Model 1, and were attenuated but remained robust and statistically significant when adding family, classmate, and teacher relationships in Model 2. Further analyses showed that the difference between those who reported a medium and a low sense of unity was statistically significant at the 0.1%-level (not presented in table).

**TABLE 3:** Results from linear regressions of psychological complaints with robust standard errors. n = 1,392. Health Behavior in School-aged Children study, Sweden, 2017/18.

	Psychological complaints					
	Crude		Model 1		Model 2	
	*b*	95% CI	*b*	95% CI	*b*	95% CI
Sense of unity						
High (ref.)	0.00	—	0.00	—	0.00	—
Medium	1.68***	1.25, 2.10	1.58***	1.15, 2.02	0.75**	0.34, 1.17
Low	2.90***	2.42, 3.38	3.05***	2.57, 3.54	1.75***	1.31, 2.20
Gender						
Boys (ref.)	0.00	—	0.00	—	0.00	—
Girls	2.45***	2.01, 2.89	2.52***	2.12, 2.93	2.17***	1.76, 2.58
Immigrant background						
No (ref.)	0.00	—	0.00	—	0.00	—
Yes	−0.74*	−1.39, −0.10	−1.05**	−1.65, −0.45	−1.01***	−1.52, −0.49
Socioeconomic position (FAS)						
High (ref.)	0.00	—	0.00	—	0.00	—
Medium	0.42	−0.07, 0.90	0.01	−0.40, 0.42	−0.06	−0.45, 0.33
Low	0.42	−0.19, 1.04	−0.15	−0.68, 0.38	−0.42	−0.93, 0.10
Family relationships						
Strong (ref.)	0.00	—			0.00	—
Intermediate	1.68***	1.17, 2.19			0.92***	0.44, 1.40
Poor	3.76***	3.24, 4.29			2.43***	1.89, 2.96
Classmate relationships						
Strong (ref.)	0.00	—			0.00	—
Intermediate	1.42***	0.98, 1.87			0.47*	0.01, 0.92
Poor	2.85***	2.31, 3.38			0.98***	0.45, 1.50
Teacher relationships						
Strong (ref.)	0.00	—			0.00	—
Intermediate	1.60***	1.10, 2.10			0.64*	0.13, 1.14
Poor	2.93***	2.32, 3.54			0.88**	0.27, 1.49

***p < 0.001 **p < 0.01 *p < 0.05.

With regards to somatic complaints, with results presented in Table 4, the crude analyses showed that both those with a medium sense of unity (*b* = 0.89, *p* < 0.001) and a low sense of unity (*b* = 1.69, *p* < 0.001) were significantly worse off compared with the group with a high sense of unity. The associations remained clear and statistically significant in Model 1. However, when adjusting for family, classmate, and teacher relationships in Model 2, the associations were attenuated and the level of somatic complaints did not differ significantly between those with a high and a medium sense of unity. Additional analyses demonstrated that the difference between those with a medium and a low sense of unity was statistically significant at the 1%-level (not presented in table).

**TABLE 4:** Results from linear regressions of somatic complaints with robust standard errors. n = 1,392. Health Behavior in School-aged Children study, Sweden, 2017/18.

	Somatic complaints					
	Crude		Model 1		Model 2	
	*b*	95% CI	*b*	95% CI	*b*	95% CI
Sense of unity						
High (ref.)	0.00	—	0.00	—	0.00	—
Medium	0.89***	0.46, 1.31	0.81***	0.40, 1.22	0.26	−0.17, 0.70
Low	1.69***	1.21, 2.17	1.78***	1.30, 2.26	0.97***	0.47, 1.47
Gender						
Boys (ref.)	0.00	—	0.00	—	0.00	—
Girls	2.01***	1.64, 2.39	2.05***	1.69, 2.42	1.83***	1.45, 2.21
Immigrant background						
No (ref.)	0.00	—	0.00	—	0.00	—
Yes	−0.16	−0.77, 0.46	−0.33	−0.94, 0.28	−0.31	−0.89, 0.27
Socioeconomic position (FAS)						
High (ref.)	0.00	—	0.00	—	0.00	—
Medium	0.41	−0.04, 0.86	0.17	−0.27, 0.60	0.15	−0.27, 0.58
Low	0.24	−0.31, 0.80	−0.16	−0.70, 0.38	−0.30	−0.83, 0.24
Family relationships						
Strong (ref.)	0.00	—			0.00	—
Intermediate	1.10***	0.65, 1.55			0.57*	0.12, 1.03
Poor	2.32***	1.84, 2.80			1.41***	0.87, 1.95
Classmate relationships						
Strong (ref.)	0.00	—			0.00	—
Intermediate	0.90***	0.48, 1.32			0.24	−0.20, 0.67
Poor	1.63***	1.13, 2.12			0.31	−0.21, 0.83
Teacher relationships						
Strong (ref.)	0.00	—			0.00	—
Intermediate	1.18***	0.75, 1.61			0.63**	0.17, 1.08
Poor	2.11***	1.60, 2.61			0.97**	0.41, 1.52

***p < 0.001 **p < 0.01 *p < 0.05.

Finally, for self-rated health, with results presented in Table 5, the crude associations with sense of unity were strong and displayed a clear gradient (medium sense of unity: OR = 3.31, *p* < 0.001; low sense of unity: OR = 7.34, *p* < 0.001). The associations remained largely the same when controlling for sociodemographic characteristics in Model 1, and were substantially reduced although still statistically significant when adding family, classmate, and teacher relationships in Model 2. Furthermore, the difference between adolescents with a medium and those with a low sense of unity was shown to be statistically significant at the 5%-level (not presented in table).

**TABLE 5:** Results from binary logistic regressions of less than good self-rated health with robust standard errors. n = 1,392. Health Behavior in School-aged Children study, Sweden, 2017/18.

	Less than good self-rated health					
	Crude		Model 1		Model 2	
	OR	95% CI	OR	95% CI	OR	95% CI
Sense of unity						
High (ref.)	1.00	—	1.00	—	1.00	—
Medium	3.31***	1.73, 6.34	3.21**	1.66, 6.23	2.04*	1.05, 3.99
Low	7.34***	4.20, 12.83	7.68***	4.19, 14.09	3.51***	1.88, 6.57
Gender						
Boys (ref.)	1.00	—	1.00	—	1.00	—
Girls	2.06***	1.39, 3.06	2.23***	1.53, 3.26	1.87**	1.25, 2.79
Immigrant background						
No (ref.)	1.00	—	1.00	—	1.00	—
Yes	0.87	0.49, 1.53	0.70	0.39, 1.25	0.75	0.42, 1.35
Socioeconomic position (FAS)						
High (ref.)	1.00	—	1.00	—	1.00	—
Medium	1.22	0.76, 1.97	0.97	0.60, 1.56	0.93	0.57, 1.51
Low	1.56	0.86, 2.86	1.10	0.58, 2.10	0.91	0.48, 1.72
Family relationships						
Strong (ref.)	1.00	—			1.00	—
Intermediate	1.59	0.80, 3.16			0.97	0.46, 2.05
Poor	6.09***	3.39, 10.92			2.65**	1.40, 5.01
Classmate relationships						
Strong (ref.)	1.00	—			1.00	—
Intermediate	2.30**	1.36, 3.89			1.42	0.85, 2.38
Poor	6.61***	3.88, 11.26			2.76**	1.52, 5.01
Teacher relationships						
Strong (ref.)	1.00	—			1.00	—
Intermediate	2.14**	1.21, 3.79			1.28	0.68, 2.38
Poor	5.65***	3.23, 9.90			1.92*	1.01, 3.66

***p < 0.001 **p < 0.01 *p < 0.05.

Regarding the sociodemographic characteristics, gender was associated with all outcomes and across all models, indicating that girls experienced worse health overall. Having an immigrant background was associated with fewer psychological complaints in the adjusted models (Table 3), whereas there was no statistically significant difference by immigration background in somatic complaints (Table 4) or in self-rated health (Table 5). No significant associations were found between socioeconomic position and any of the outcomes.

The variables measuring family, classmate, and teacher relationships varied in their association with the outcome variables. In the crude analyses, reporting weaker relationships in each of these domains was associated with worse health for all outcomes. The associations were attenuated in the fully adjusted models but remained statistically significant for family relationships and teacher relationships in all three instances as well as for classmate relationships in relation to psychological complaints and less than good self-rated health.

None of the tested interactions proved to significantly improve the model fit (not presented in table). We also performed gender-stratified analyses for all three health outcomes, assessing both crude and adjusted associations (see Supplementary Tables A1–A3). These show that the associations between sense of unity and health are stronger and more robust for girls across all outcomes.

Finally, several additional checks were performed by running a series of linear and binary logistic regressions to verify the robustness of the results. First, each psychological and somatic complaint was analyzed separately (Supplementary Table A4), and second, different partitions of the sense of unity variable as well as the continuous measure were tested (Supplementary Table A5). The patterns of these analyses strengthen our overall results, indicating a gradual association between sense of unity and health.

## Discussion

The current study examined the links between sense of unity, defined as the positive feeling of being part of a larger social structure [15], and aspects of self-reported health among 15-year-olds in Sweden.

Analyses of data from the Swedish Health Behavior in School-aged Children (HBSC) survey of 2017/18 showed that, overall, the participants reported a high level of sense of unity. No difference in sense of unity was detected between genders, but adolescents with an immigrant background reported lower levels than those without such background, and adolescents with lower family affluence reported lower levels compared to those with higher family affluence. Sense of unity was also positively associated with family, classmate, and teacher relationships.

Lower levels of sense of unity were associated with higher levels of psychological and somatic complaints, and with an increased likelihood of less than good self-rated health. These associations remained strong and robust even when adjusting for family, classmate, and teacher relationships and across various categorizations of sense of unity and of the outcome variables, suggesting that sense of unity is a feature with independent relevance for health. The fact that the associations were clear and robust across all three health indicators, as well as showing a clear gradient for two out of three measures (namely, psychological complaints and less than good self-rated health), strengthens this interpretation. Moreover, the lack of statistically significant interaction effects for sense of unity and the control variables indicates that the findings are valid across groups of adolescents. The clear and robust links between sense of unity and self-reported health reflect prior research from Australia showing that general belonging was inversely associated with depressive symptoms in a community sample of 13–17-year olds [13] as well as in a sample of gay, lesbian, and bisexual youth [14]. The current study corroborates and extends these findings by demonstrating similar patterns in a national sample of Swedish 15-year-olds. The results are also in line with findings from previous studies that have linked adolescent health to sense of community [19, 20] and to connectedness [21]. However, while sense of community refers to belongingness to a specific community, and connectedness commonly to tangible social relationships, sense of unity differs from these concepts due to its focus on feelings of belonging to society in a wider, general sense. Hence, the current study adds to the body of previous research by indicating that feelings of being part of, and contributing to, the larger social fabric in which they are embedded may be an important social determinant for adolescent health, over and above the effect of tangible social relationships in the family and at school. Suggested mechanisms for why sense of community and connectedness protect against adverse mental health include the construction of a social identity, less feelings of loneliness, perceptions of belonging, closeness, and support, and exposure to positive modeling [22, 26, 42–44]. These, in addition to the provision of a sense of common good [15], are all possible ways in which sense of unity might operate as well, albeit specifically in relation to positive feelings of belongingness to society at large.

While this study did not report any difference in sense of unity between boys and girls, earlier studies examining gender differences in related concepts such as sense of community and connectedness have produced inconsistent results [23, 24, 26, 45, 46]. It is possible that varying contexts, concepts, and measurements are linked with different gender specific advantages in regards to sense of unity and similar concepts, but there does not seem to be any general pattern that favors either boys or girls. Although we did not find any statistically significant interaction effects, gender-stratified analyses revealed that the associations between sense of unity and health were more pronounced for girls. This may indicate that there exist gender differences in the correlates of sense of unity. Our finding that adolescents with an immigrant background reported lower levels of sense of unity compared with those without such a background reflects findings from prior studies on sense of community and connectedness [27, 28]. It has also been suggested that sense of community is especially important for immigrants’ health [27] and that connectedness can aid in the acculturation process [47]. Sense of unity may therefore be especially important for adolescents with an immigrant background, and may reflect how well these individuals are integrated in the host society. The fact that this category reported lower levels of sense of unity in the current study is of concern. With regards to socioeconomic position, prior studies of adolescents have demonstrated an inverse association with sense of community [26] as well as with connectedness [23]. Putnam et al. [48] present evidence for a widening class gap, in that American adolescents from working class backgrounds have become more disconnected from societal institutions since the 1970s. Sweden has faced a rapid inequality growth the last decades [49] and if this leads to disparities in sense of unity by socioeconomic background, the implications for health could be severe.

Our finding that sense of unity was positively and clearly associated with family, classmate, and teacher relationships suggests that tangible social relationships in the family and at school could promote more general feelings of belonging to society at large. The fact that the associations between sense of unity and the relationship variables were not perfect suggests that they capture differing concepts. Nonetheless, it should be acknowledged that the high correlations may also indicate that the constructs are not essentially different but may have some borderline conceptual overlap. More research is needed to both theoretically distinguish sense of unity from other similar concepts and to empirically asses the links between sense of unity and other measures within the realm of the social world such as relationships in specific domains.

The main contribution of this study is the use of sense of unity as a new measure to analyze the association between adolescents’ feelings of belongingness to society and their self-reported health. The use of three different health outcomes strengthens the interpretation that sense of unity is associated with adolescent health to some degree. Another benefit concerns the fact that the data were based on a nationally representative sample, and that the classroom surveys implied a high participation rate at the student-level, although the non-participation at the school-level was more substantial [37, 38]. Since we lack information about which schools participated in the study, there are however limited possibilities of investigating to what extent there is systematic response bias at the school-level. Another limitation concerns the cross-sectional nature of the data, which lessens the validity of causal inferences. While sense of unity might affect health, the opposite may be true as well, i.e., those with poor health could be more prone to report lower levels of sense of unity due to their current ill state. It is also possible that some common underlying reason, e.g., negative affectivity [50] may make an individual prone to report both a low sense of unity and poor health. Future studies should use longitudinal data to disentangle the direction of the association.

We also identify several other possible avenues for further inquiry on sense of unity. First, to be able to generalize our findings, future research should analyze sense of unity and its correlates in other populations (e.g., in other geographical settings and in other age groups). Furthermore, examining the connection between sense of unity and other types of outcomes, health risk behaviors or delinquency [51] for instance, may be promising. Yet another relevant task for future research is to follow individuals who report a low sense of unity in adolescence across the life course, to assess if they have an increased risk of adverse health (and/or other types of disadvantages) in adulthood. Lastly, more research is needed to study the origins and mechanisms behind sense of unity and its distribution across different social groups, and subsequently, how sense of unity can be strengthened by different societal institutions.

In conclusion, the present study showed that sense of unity was associated with self-reported health among adolescents, even when controlling for family, classmate, and teacher relationships, indicating that sense of unity may be an important social determinant for adolescent health. The findings suggest that one way to prevent, or at least mitigate, adverse health is to promote sense of unity among youth. As for public health policies emanating from this knowledge, schools should be an arena of utmost importance as they can provide a compensatory role for disadvantaged youth. In this case, adolescents with a low sense of unity, and especially those from groups that are less integrated in society, might be supplied with resources that strengthen their sense of unity [25]. Another area with potential is adolescents’ leisure time activities. Participating in structured and meaningful extracurricular activities could be a way for youth to experience a stronger sense of unity [19]. Regardless of area or type of intervention it is important to not only focus on the level of sense of unity in the present moment but also on adolescents’ ability to maintain these feelings over time, and into adulthood [23]. Still, the participants reported a high level of sense of unity overall. The level of sense of unity however differed by sociodemographic characteristics, which is noteworthy and encourages further investigations.

## Data Availability

The data analyzed in this study is subject to the following licenses/restrictions: The Swedish HBSC data of 2017/18 can be applied for at the Public Health Agency of Sweden. Data from previous waves in Sweden and in other participating countries is available at: https://www.uib.no/en/hbscdata. Requests to access these datasets should be directed to Public Health Agency of Sweden.

## Abbreviations

95% CI, 95% confidence interval; b, regression coefficient; HBSC, health behavior in school-aged children; OR, odds ratio; ref, reference category.
