# Supplementary material for: Sense of Unity and Self-Reported Health Among 15-year-Olds: Findings From the Swedish 2017/18 Health Behavior in School-Aged Children Study
Source: Int J Public Health. 2021 Apr 20;66:621964. doi: 10.3389/ijph.2021.621964 (PMC8565282; doi:10.3389/ijph.2021.621964)
Supplement: Supplementary file 1 [file Table1.DOC]

Supplementary Material

**Table A1.** Results from linear regressions of psychological complaints with robust standard errors, stratified by gender. Boys: n=646; girls: n=746

|  | Psychological complaints | | | | | | | | | | | |
| --- | --- | --- | --- | --- | --- | --- | --- | --- | --- | --- | --- | --- |
|  | Boys | | | | | | Girls | | | | | |
|  | Crude | | Model 1 | | Model 2 | | Crude | | Model 1 | | Model 2 | |
|  | *b* | 95% CI | *b* | 95% CI | *b* | 95% CI | *b* | 95% CI | *b* | 95% CI | *b* | 95% CI |
| Sense of unity |  |  |  |  |  |  |  |  |  |  |  |  |
| High (ref.) | 0.00 | - | 0.00 | - | 0.00 | - | 0.00 | - | 0.00 | - | 0.00 | - |
| Medium | 1.59*** | 0.90, 2.20 | 1.63*** | 0.99, 2.27 | 0.67* | 0.06, 1.29 | 1.61*** | 1.08, 2.14 | 1.57*** | 1.03, 2.11 | 0.79** | 1.44, 2.75 |
| Low | 2.62*** | 1.87, 3.37 | 2.76*** | 2.02, 3.50 | 1.34** | 0.65, 2.03 | 3.27*** | 2.68, 3.86 | 3.32*** | 2.70, 3.94 | 2.10*** | 0.21, 1.37 |
| Immigrant background |  |  |  |  |  |  |  |  |  |  |  |  |
| No (ref.) | 0.00 | - | 0.00 | - | 0.00 | - | 0.00 | - | 0.00 | - | 0.00 | - |
| Yes | -0.93* | -1.70, -0.16 | -1.07** | -1.79, -0.35 | -1.02** | -1.69, -0.36 | -0.82 | -1.64, 0.01 | -1.04* | -1.83, -0.24 | -1.02** | -1.71, -0.34 |
| Socioeconomic position (FAS) |  |  |  |  |  |  |  |  |  |  |  |  |
| High (ref.) | 0.00 | - | 0.00 | - | 0.00 | - | 0.00 | - | 0.00 | - | 0.00 | - |
| Medium | -0.25 | -0.87, 0.38 | -0.51 | -1.15, 0.13 | -0.41 | -1.00, 0.17 | 0.87* | 0.12, 1.61 | 0.48 | -0.17, 1.13 | 0.27 | -0.32, 0.86 |
| Low | -0.04 | -0.86, 0.77 | -0.48 | -1.23, 0.27 | -0.75* | -1.45, -0.06 | 0.68 | -0.15, 1.52 | 0.16 | -0.60, 0.92 | -0.07 | -0.80, 0.66 |
| Family relationships |  |  |  |  |  |  |  |  |  |  |  |  |
| Strong (ref.) | 0.00 | - |  |  | 0.00 | - | 0.00 | - |  |  | 0.00 | - |
| Intermediate | 1.66*** | 0.99, 2.33 |  |  | 1.09** | 0.45, 1.73 | 1.36** | 0.61, 2.10 |  |  | 0.80* | 0.06, 1.54 |
| Poor | 3.25*** | 2.56, 3.95 |  |  | 2.11*** | 1.41, 2.80 | 3.69*** | 3.02, 4.36 |  |  | 2.63*** | 1.88, 3.38 |
| Classmate relationships |  |  |  |  |  |  |  |  |  |  |  |  |
| Strong (ref.) | 0.00 | - |  |  | 0.00 | - | 0.00 | - |  |  | 0.00 | - |
| Intermediate | 1.61*** | 1.04, 2.18 |  |  | 0.82** | 0.22, 1.42 | 1.02** | 0.32, 1.72 |  |  | 0.12 | -0.53, 0.77 |
| Poor | 2.85*** | 2.03, 3.67 |  |  | 1.39** | 0.62, 2.15 | 2.20*** | 1.45, 2.94 |  |  | 0.63 | -0.06, 1.33 |
| Teacher relationships |  |  |  |  |  |  |  |  |  |  |  |  |
| Strong (ref.) | 0.00 | - |  |  | 0.00 | - | 0.00 | - |  |  | 0.00 | - |
| Intermediate | 1.57*** | 0.99, 2.15 |  |  | 0.70* | 0.11, 1.28 | 1.20** | 0.48, 1.92 |  |  | 0.53 | -0.02, 1.46 |
| Poor | 2.77*** | 1.90, 3.63 |  |  | 1.13* | 0.23, 2.04 | 2.43*** | 1.68, 3.19 |  |  | 0.72 | -0.21, 1.28 |

***p<0.001 **p<0.01 *p<0.05

**Table A2.** Results from linear regressions of somatic complaints with robust standard errors, stratified by gender. Boys: n=646; girls: n=746

|  | Somatic complaints | | | | | | | | | | | |
| --- | --- | --- | --- | --- | --- | --- | --- | --- | --- | --- | --- | --- |
|  | Boys | | | | | | Girls | | | | | |
|  | Crude | | Model 1 | | Model 2 | | Crude | | Model 1 | | Model 2 | |
|  | *b* | 95% CI | *b* | 95% CI | *b* | 95% CI | *b* | 95% CI | *b* | 95% CI | *b* | 95% CI |
| Sense of unity |  |  |  |  |  |  |  |  |  |  |  |  |
| High (ref.) | 0.00 | - | 0.00 | - | 0.00 | - | 0.00 | - | 0.00 | - | 0.00 | - |
| Medium | 0.68* | 0.11, 1.25 | 0.72* | 0.15, 1.30 | 0.10 | -0.47, 0.66 | 0.91** | 0.30, 1.52 | 0.88** | 0.26, 1.49 | 0.34 | -0.35, 1.04 |
| Low | 1.54*** | 0.89, 2.20 | 1.65*** | 0.98, 2.31 | 0.77* | 0.09, 1.45 | 1.90*** | 1.20, 2.60 | 1.90*** | 1.19, 2.62 | 1.12** | 0.31, 1.94 |
| Immigrant background |  |  |  |  |  |  |  |  |  |  |  |  |
| No (ref.) | 0.00 | - | 0.00 | - | 0.00 | - | 0.00 | - | 0.00 | - | 0.00 | - |
| Yes | -0.27 | -0.97, 0.43 | -0.32 | -1.01, 0.38 | -0.31 | -0.99, 0.37 | -0.24 | -1.02, 0.53 | -0.35 | -1.16, 0.46 | -0.35 | -1.12, 0.43 |
| Socioeconomic position (FAS) |  |  |  |  |  |  |  |  |  |  |  |  |
| High (ref.) | 0.00 | - | 0.00 | - | 0.00 | - | 0.00 | - | 0.00 | - | 0.00 | - |
| Medium | -0.04 | -0.67, 0.59 | -0.19 | -0.83, 0.46 | -0.09 | -0.70, 0.51 | 0.71* | 0.07, 1.34 | 0.49 | -0.14, 1.11 | 0.40 | -0.23, 1.02 |
| Low | -0.19 | -0.91, 0.53 | -0.49 | -1.19, 0.20 | -0.65 | -1.31, 0.01 | 0.50 | -0.21, 1.20 | 0.14 | -0.59, 0.87 | 0.03 | -0.71, 0.77 |
| Family relationships |  |  |  |  |  |  |  |  |  |  |  |  |
| Strong (ref.) | 0.00 | - |  |  | 0.00 | - | 0.00 | - |  |  | 0.00 | - |
| Intermediate | 0.83** | 0.26, 1.40 |  |  | 0.46 | -0.14, 1.06 | 1.07** | 0.44, 1.70 |  |  | 0.69* | 0.01, 1.37 |
| Poor | 1.87*** | 1.21, 2.53 |  |  | 1.13** | 0.39, 1.88 | 2.31*** | 1.71, 2.91 |  |  | 1.64*** | 0.92, 2.37 |
| Classmate relationships |  |  |  |  |  |  |  |  |  |  |  |  |
| Strong (ref.) | 0.00 | - |  |  | 0.00 | - | 0.00 | - |  |  | 0.00 | - |
| Intermediate | 1.14*** | 0.58, 1.70 |  |  | 0.69* | 0.13, 1.25 | 0.47 | -0.16, 1.11 |  |  | -0.00 | -0.71, 0.70 |
| Poor | 1.59*** | 0.86, 2.32 |  |  | 0.66 | -0.09, 1.40 | 1.09** | 0.43, 1.75 |  |  | -0.18 | -0.81, 0.45 |
| Teacher relationships |  |  |  |  |  |  |  |  |  |  |  |  |
| Strong (ref.) | 0.00 | - |  |  | 0.00 | - | 0.00 | - |  |  | 0.00 | - |
| Intermediate | 1.07*** | 0.52, 1.61 |  |  | 0.62* | 0.04, 1.20 | 0.94** | 0.35, 1.54 |  |  | 0.60 | -0.04, 1.24 |
| Poor | 1.90*** | 1.18, 2.61 |  |  | 1.06** | 0.30, 1.82 | 1.78*** | 1.05, 2.50 |  |  | 0.92* | 0.09, 1.74 |

***p<0.001 **p<0.01 *p<0.05

**Table A3.** Results from binary logistic regressions of less than good self-rated health with robust standard errors, stratified by gender. Boys: n=646; girls: n=746

|  | Less than good self-rated health | | | | | | | | | | | |
| --- | --- | --- | --- | --- | --- | --- | --- | --- | --- | --- | --- | --- |
|  | Boys | | | | | | Girls | | | | | |
|  | Crude | | Model 1 | | Model 2 | | Crude | | Model 1 | | Model 2 | |
|  | OR | 95% CI | OR | 95% CI | OR | 95% CI | OR | 95% CI | OR | 95% CI | OR | 95% CI |
| Sense of unity |  |  |  |  |  |  |  |  |  |  |  |  |
| High (ref.) | 1.00 |  | 1.00 |  | 1.00 |  | 1.00 |  | 1.00 |  | 1.00 |  |
| Medium | 2.25 | 0.80, 6.34 | 2.19 | 0.78, 6.15 | 1.41 | 0.48, 4.16 | 4.11** | 1.66, 10.20 | 4.19** | 1.66, 10.59 | 2.70* | 1.04, 7.02 |
| Low | 4.05** | 1.62, 10.17 | 3.71** | 1.49, 9.21 | 1.38 | 0.49, 3.90 | 10.96*** | 4.66, 25.77 | 11.71*** | 4.66, 29.41 | 5.82*** | 2.23, 15.18 |
| Immigrant background |  |  |  |  |  |  |  |  |  |  |  |  |
| No (ref.) | 1.00 |  | 1.00 |  | 1.00 |  | 1.00 |  | 1.00 |  | 1.00 |  |
| Yes | 0.79 | 0.34, 1.84 | 0.65 | 0.26, 1.61 | 0.67 | 0.27, 1.66 | 0.86 | 0.45, 1.63 | 0.74 | 0.38, 1.45 | 0.81 | 0.41, 1.60 |
| Socioeconomic position (FAS) |  |  |  |  |  |  |  |  |  |  |  |  |
| High (ref.) | 1.00 |  | 1.00 |  | 1.00 |  | 1.00 |  | 1.00 |  | 1.00 |  |
| Medium | 1.05 | 0.48, 2.31 | 0.94 | 0.42, 2.09 | 0.93 | 0.40, 2.19 | 1.25 | 0.72, 2.16 | 0.95 | 0.55, 1.63 | 0.91 | 0.52, 1.61 |
| Low | 2.24 | 0.84, 5.97 | 1.85 | 0.66, 5.17 | 1.49 | 0.50, 4.44 | 1.25 | 0.62, 2.52 | 0.82 | 0.40, 1.69 | 0.70 | 0.34, 1.45 |
| Family relationships |  |  |  |  |  |  |  |  |  |  |  |  |
| Strong (ref.) | 1.00 |  |  |  | 1.00 |  | 1.00 |  |  |  | 1.00 |  |
| Intermediate | 0.85 | 0.25, 2.84 |  |  | 0.59 | 0.19, 1.84 | 2.10 | 0.87, 5.05 |  |  | 1.37 | 0.52, 3.59 |
| Poor | 4.44** | 1.91, 10.31 |  |  | 1.91 | 0.69, 5.26 | 7.07*** | 3.17, 15.77 |  |  | 3.41** | 1.44, 8.10 |
| Classmate relationships |  |  |  |  |  |  |  |  |  |  |  |  |
| Strong (ref.) | 1.00 |  |  |  | 1.00 |  | 1.00 |  |  |  | 1.00 |  |
| Intermediate | 2.31 | 0.89, 5.96 |  |  | 1.74 | 0.64, 4.75 | 2.19* | 1.10, 4.35 |  |  | 1.34 | 0.63, 2.82 |
| Poor | 9.44*** | 3.38, 26.39 |  |  | 4.85** | 1.58, 14.91 | 4.95*** | 2.67, 9.18 |  |  | 2.25* | 1.05, 4.84 |
| Teacher relationships |  |  |  |  |  |  |  |  |  |  |  |  |
| Strong (ref.) | 1.00 |  |  |  | 1.00 |  | 1.00 |  |  |  | 1.00 |  |
| Intermediate | 3.01 | 0.92, 9.84 |  |  | 1.77 | 0.55, 5.73 | 1.65 | 0.78, 3.49 |  |  | 1.04 | 0.45, 2.42 |
| Poor | 7.83*** | 2.65, 23.12 |  |  | 2.83 | 0.85, 9.46 | 4.25*** | 2.08, 8.71 |  |  | 1.52 | 0.62, 3.71 |

***p<0.001 **p<0.01 *p<0.05

**Table A4.** Results from binary logistic regressions with robust standard errors, analyzing specific psychological and somatic complaints more than weekly. All models adjusted for gender, immigration background, socioeconomic position, and family, classmate, and teacher relationships. The percentages refer to the proportions of participants with each complaint more than weekly. n=1,392

|  | Psychological complaints | | | | Somatic complaints | | | |
| --- | --- | --- | --- | --- | --- | --- | --- | --- |
|  | Feeling low | Irritability or bad temper | Feeling nervous | Sleep difficulties | Headache | Stomach ache | Backache | Feeling dizzy |
|  | (30.1%) | (45.6%) | (28.5%) | (30.2%) | (24.7%) | (17.6%) | (16.3%) | (13.2%) |
|  |  |  |  |  |  |  |  |  |
|  | OR | OR | OR | OR | OR | OR | OR | OR |
| Sense of unity |  |  |  |  |  |  |  |  |
| High (ref.) | 1.00 | 1.00 | 1.00 | 1.00 | 1.00 | 1.00 | 1.00 | 1.00 |
| Medium | 1.98** | 1.33* | 1.28 | 1.03 | 0.97 | 0.87 | 0.77 | 1.24 |
| Low | 3.44*** | 1.99*** | 1.67** | 2.00*** | 1.38 | 1.70** | 1.24 | 2.27*** |

***p<0.001 **p<0.01 *p<0.05

**Table A5.** Results from OLS and binary logistic regressions with robust standard errors, with alternative categorizations of sense of unity. All models adjusted for gender, immigration background, socioeconomic position, and family, classmate, and teacher relationships. The percentages refer to the proportions of participants with different levels of sense of unity according to each categorization. For the continuous measure of sense of unity, the range is presented. n=1,392

|  |  | Psychological complaints | Somatic complaints | Less than good self-rated health |
| --- | --- | --- | --- | --- |
|  |  | *b* | *b* | OR |
| Sense of unity (3 categories) |  |  |  |  |
| Scores 34 to 40 (ref.) | (45.6%) | 0.00 | 0.00 | 1.00 |
| Scores 25 to <34 | (45.2%) | 1.01*** | 0.41* | 2.35** |
| Scores 8 to 24 | (9.3%) | 2.10*** | 1.72*** | 4.32*** |
| Sense of unity (4 categories) |  |  |  |  |
| Scores >36 to 40 (ref.) | (22.6%) | 0.00 | 0.00 | 1.00 |
| Scores 33 to 36 | (29.2%) | 0.74** | 0.60* | 0.79 |
| Scores >29 to 32 | (22.2%) | 1.23*** | 0.70** | 2.70* |
| Scores 8 to 29 | (26.0%) | 2.19*** | 1.54*** | 2.77* |
| Sense of unity (5 categories) |  |  |  |  |
| Scores 37 to 40 (ref.) | (22.1%) | 0.00 | 0.00 | 1.00 |
| Scores >34 to <37 | (15.4%) | 0.58 | 0.72* | 0.67 |
| Scores 32 to 34 | (24.0%) | 1.04*** | 0.45 | 1.53 |
| Scores 28 to 31 | (20.9%) | 1.65*** | 1.05* | 2.40 |
| Scores 8 to <28 | (17.6%) | 2.33*** | 1.67*** | 2.89* |
| Sense of unity (continuous) | 8-40 | -0.12*** | -0.08*** | 0.93*** |

***p<0.001 **p<0.01 *p<0.05
